# Paraneoplastic Syndromes in Patients with Keratinocyte Skin Cancer

**DOI:** 10.3390/cancers14010249

**Published:** 2022-01-04

**Authors:** Christoforos Vlachos, Chrysanthi Tziortzioti, Ioannis D. Bassukas

**Affiliations:** 1 Division of Skin and Venereal Diseases, School of Health Sciences, Faculty of Medicine, University of Ioannina, 45100 Ioannina, Greece; christofvlachos@uoi.gr; 2 Dermatology Clinic, General University Hospital of Ioannina, 45100 Ioannina, Greece; sissitziortzioti@gmail.com

**Keywords:** paraneoplasia, keratinocyte skin cancer, basal cell carcinoma, squamous cell carcinoma, adnexal skin tumours, hidradenitis suppurativa

## Abstract

**Simple Summary:**

The aim of the present review is to compile and evaluate the literature data on paraneoplastic syndromes (PNS) associated with keratinocyte skin cancer (KSC). Forty relevant entries were assembled, reporting a total of 41 PNS cases associated with a KSC (34 male). No review paper compiling this topic was found. Six distinct PNS entities were identified, and malignancy associated hypercalcemia (MAH; 78%), anemia (10%) and Bazex syndrome (5%) were the most frequently reported among them. 85% of the PNS were reported in association with SCC, 10% with BCC, and the rest with adnexal tumors. The median age of the patients at the time of PNS diagnosis was 58 years (range: five–83 years). KSC predisposing conditions, as scars (22%) or hidradenitis suppurativa (20%), were reported in >70% of the PNS cases. In conclusion, PNS are rarely reported in association with KSC, possibly reflecting a limited capacity of KSC to provoke overt PNS.

**Abstract:**

A variety of well-characterized cutaneous paraneoplastic syndromes (PNS) are diagnosed during internal malignancies; however, the spectrum of keratinocyte skin neoplasms (KSC) related to PNS is still obscure. The aim of the present review is to compile and evaluate the literature data on PNS associated with a keratinocyte skin neoplasm (KSC). Employing Pubmed, MEDLINE was searched for KSC-associated PNS reports. Forty relevant entries were assembled, reporting a total of 41 PNS cases associated with a KSC (34 male). No review paper compiling this topic was found. Six distinct PNS entities were identified, and malignancy associated hypercalcemia (MAH; 78%), anemia (10%) and Bazex syndrome (5%) were the most frequently reported among them. 85% of the PNS were reported in association with SCC, 10% with BCC, and the rest with adnexal tumors. The median age of the patients at the time of PNS diagnosis was 58 years (range: five–83 years). In most cases the PNS was diagnosed either concurrently or after the KSC diagnosis. KSC predisposing conditions, as scars (22%) or hidradenitis suppurativa (20%), were reported in >70% of the PNS cases. Most PNS resolved after KSC treatment. In conclusion, PNS of a rather limited spectrum of entities are reported in association with KSC. They also seem to be rare, possibly reflecting a limited capacity of KSC to provoke overt PNS.

## 1. Introduction

Paraneoplastic syndromes (PNS) are nonmetastatic tumor-associated findings caused by malignancies in remote body sites [1,2]. PNS are found in a considerable fraction of tumor patients and may primarily affect the neuromuscular/musculoskeletal, cardiovascular, cutaneous, hematologic, gastrointestinal, endocrine or renal systems [2]. Moreover, in some cases PNS may be even the first or most prominent manifestation of an underlying cancer, so their timely identification can guide the search for a still unrecognized malignancy. From a pathophysiologic point of view, most PNS are attributed to either endocrine phenomena (induced by tumor secreted biomolecules) or auto-immunity mediated immunological mechanisms [3,4]. Although the skin is a frequent target organ of PNS [5,6], PNS have been rather rarely reported in the course of skin neoplasms, even in relation to the biologically more aggressive ones, like malignant melanoma and Merkel cell carcinoma [7,8]. Herein, we compile the reported evidence of PNS associated with the growth of keratinocyte skin cancers (KSC). Our aim was to provide a typology of the spectrum of these PNS as an aid for the everyday clinical practice and as a basis for future comparative and mechanistic studies. To do so, we conducted a focused literature search and present our findings in the form of a research synthesis.

## 2. Materials and Methods

**Figure 1:**
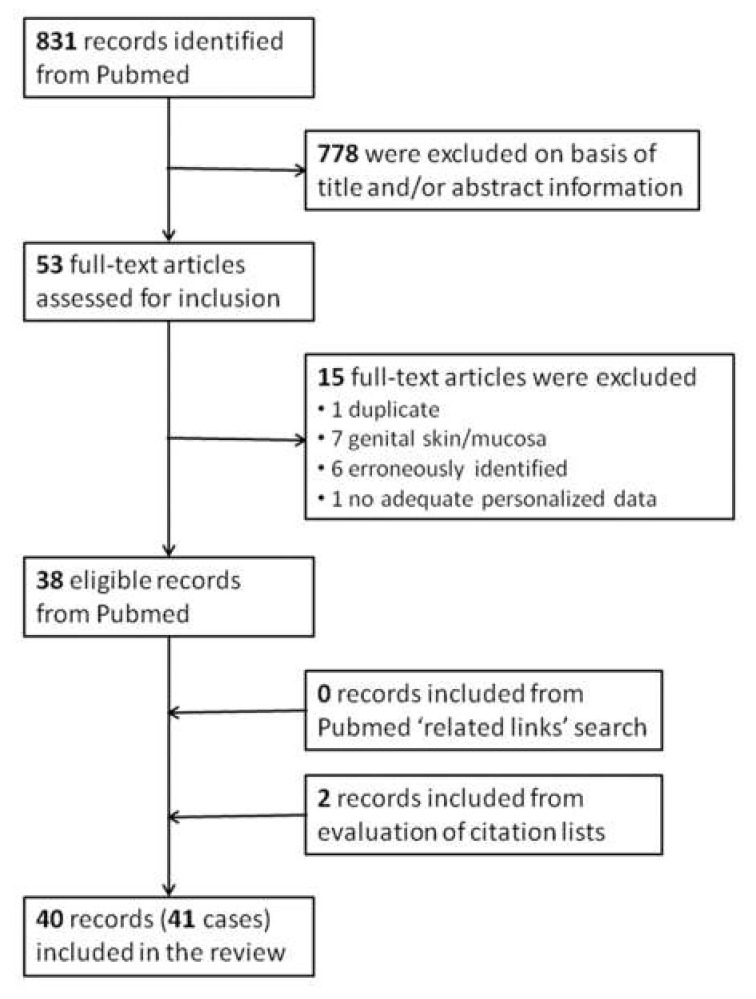
Literature search: Flowchart of publicationsrelated to PNS cases in keratinocyte skin cancers selection process.

Only PNS attributed to KSC were included in this compilation, i.e., cutaneous squamous cell carcinomas (cSCC), basal cell carcinomas (BCC) and skin adnexal neoplasms. MEDLINE was searched using Pubmed (8 May 2021) for publications reporting PNS caused by KSC ({C}) with following search strategy: {C} = {A} AND {B}, with {A} = {[(basal cell carcinoma) OR (BCC)] OR [[(squamous cell carcinoma) OR (scc)] AND [(cutaneous) OR (skin)]] OR [keratinocyt* skin cancer] OR [(skin cancer) NOT (melano*)]} and {B} = {paraneopl*} OR {BN}, with {BN} = {B1} OR {B2} OR… OR {Bi} OR … OR {B43}. Bi = {text i} represents a paraneoplastic syndrome in text form, with Bi the i^th^ of the 43 (“most widely accepted”) PNS (i = 1-43), listed in the review article by Bilynsky et al. [2]. All authors analyzed the assembled material independently for reports of PNS and KSC (including stage and histology) as the only malignant disease in the history of the patient and results were merged. By evaluating the title/abstract information of the initially returned 831 papers we localized 53 articles with potentially relevant content for full-text search (Figure 1). Of them, 15 were excluded (one was a duplicate publication, seven were cases of penile SCC, six were erroneously identified as reporting PNS in KSC and one reported no adequately personalized data) leaving to 39 papers reporting a total of 44 PNS cases associated with KSC [9,10,11,12,13,14,15,16,17,18,19,20,21,22,23,24,25,26,27,28,29,30,31,32,33,34,35,36,37,38,39,40,41,42,43,44,45,46,47]. One paper ([47]) referred to five cases with relevant findings, however without any personalized data, and was excluded from this compilation, resulting in 38 papers reporting 39 relevant cases. An additional search of the selected papers for similar material in MEDLINE via the Pubmed option “related links” did not yield any additional relevant material. A search based on the references lists of the selected publications yielded two additional relevant non-MEDLINE included publications [48,49], leading to a total of 40 papers reporting 41 PNS cases associated with KSC. The KSC were classified as primary, locally relapsed (after definite therapy and no evidence of metastases) or metastatic (with locoregional or distant metastases). Core clinical data of the 41 identified PNS cases in patients with KSC are compiled in Table 1 and are summarized by descriptive statistics.

## 3. Results

No review paper compiling the topic of KSC-associated PNS was found. Our literature search yielded only papers reporting solitary cases (40 papers reporting 41 PNS cases associated with KSC, Table 1). The core clinical features of the patients are compiled in Table 2 and stratified according to patients’ gender and KSC type in Tables S1 and S2 (Supplementary Materials), respectively. Of the 41 patients with KSC-associated PNS, 34 were male (83%) and only seven were (17%) female. Thirty-five PNS cases (85%) were associated with cSCC, four with BCC (10%; one of them with squamous metaplasia) and two (5% each) with other KSC (trichilemmal carcinoma and pilomatrixoma; compare Table S2, Supplementary Materials).

Six distinct PNS entities (excluding variations) were reported (Table 2). Malignancy-associated hypercalcemia (MAH) was the most reported PNS (32/41 cases, 78% of all reported PNS cases: Table 2). MAH was primarily associated with cSCC: 30/35 cSCC cases (85%, Table 2) and 30/32 (94%, Table S2, Supplementary Materials) of the MAH cases. In two additional cases MAH was also described in patients with a rarer KSC variant, a pilomatrixoma and a trichilemmal carcinoma. It is worth noting that no cases of MAH were reported in association with a BCC. The core laboratory findings of the MAH cases are displayed in Table S3 (Supplementary Materials) and are compiled in Table 3. In five cases MAH was accompanied by leukocytosis (hypercalcemia-leukocytosis syndrome; Table S3, Supplementary Materials). Hypercalcemia was severe in most (19/32, 60%), mild in four and moderate in nine cases (Table 3). Parathormone related protein (PTHrP) was increased in most cases with available data (20/21 cases), while parathormone (PTH) was either decreased (15/28 cases) or within normal range (13/28 cases). Phosphorus and 1.25(OH)_2_D_3_ serum levels were in most cases either normal or decreased.

Other PNS included anemia in four patients (two cases of pure red cell aplasia), Bazex syndrome in two cSCC cases and three other syndromes (antiphospholipid syndrome, inflammatory arthralgias and neuropathy; Table 1 and Table 2). Notably, different KSC entities tended to associate with different PNS: KSC of squamous phenotype (cSCC and adnexal tumors) with MAH (32/32 MAH cases) and BCC with anemia (3/4 cases, Table S2, Supplementary Materials).

Considering the timing of PNS manifestation, PNS was diagnosed in most cases (38/41 patients) either concurrently or after the KSC (Table 2). A KSC predisposing condition, local or systemic in the context of a genodermatosis, was reported in >70% of the PNS cases with available information (23/32 cases; Table 2). In 4/23 cases with a predisposing factor (17%) the predisposing condition was a genodermatosis and in 19/23 cases (83%) some acquired condition (Table 2). Among the latter, scars (9 cases, 22%) and hidradenitis suppurativa (8 cases, 20%) were the most frequently associated conditions (Table 2). Finally, in most cases with available information, the PNS resolved after KSC treatment (25/38 cases, 66%, Table 2)

The median age of the patients at the time of PNS diagnosis was 58 years (range: five-83 years; Table 4). Overall, patients with any condition predisposing to KSC were younger at the time of PNS diagnosis (median age: 50.0, range five–74 years) compared to the rest (median age: 68.5, range 25–83 years; Table 4). This was particularly true for patients with a genodermatosis (compare Table 1).

## 4. Discussion

Malignancy associated hypercalcemia (MAH) [50,51,52] was the PNS most frequently reported in patients with KSC. Serum calcium levels are rigorously regulated in health (normal range: 8.2–10.6 mg/dL), and depending on the degree of hypercalcemia a plethora of clinical findings may result, ranging from non-specific symptoms like asthenia, nausea, and loss of appetite for mild hypercalcemia to more specific presentations for calcium levels >12.0 mg/dL (confusion, lethargy, coma) and finally to shock and death for extremely high serum calcium levels (>18.0 mg/dL) [50]. Immediate medical attention is necessary in these cases to reduce serum calcium levels, restore the glomerular filtration rate, and treat the underlying KSC to allow for the resolution of the crisis.

Almost all KSC-associated MAH cases presented with laboratory profiles consistent with the diagnosis of humoral hypercalcemia of malignancy (HHM), a PNS seen in up to 20% of cancer patients in the course of their disease [50,51,52]. HHM is caused by ectopic secretion of PTHrP, an immunologically distinct member of the PTH polypeptides hormone family [51] that acts through the PTH receptor to induce ubiquitous bone resorption and to reduce renal calcium clearance and phosphorus reabsorption. Increased PTHrP serum levels were also reported in most MAH cases (including two hypercalcemia-leukocytosis cases) with relevant information in the presently compiled material (Table 3). PTHrP is secreted by normal and malignant human keratinocytes and seems to act as a paracrine factor during normal growth and differentiation of the epidermis [53]. It is worth noting that the scarcity of reports identified herein seems to confirm the clinical experience that hypercalcemia is only occasionally diagnosed in association with SCC of epidermal origin, and this contrasts with the fact that SCC arising in extracutaneous sites accounts for a substantial fraction of all MAH cases [51,54]. This latter finding is in accordance with the conclusion of a former study that hypercalcemia is rare in patients with KSC [47]. Remarkably, although a cSCC complicating a severe hidradenitis suppurativa case is a rather exceptional event, ref. [10] a substantial fraction of the present PNS cases (8/41 cases), particularly MAH (7/41 or 17% of all cases), were observed within this patient setting. As MAH may develop on the background of concurrency with some other hypercalcemia-predisposing condition, like a granulomatous disease [50], it is worth inquiring whether a granulomatous hidradenitis suppurativa variant [55] underlies the development of this PNS.

With four cases, anemia was the second most frequent PNS reported in patients with KSC. Anemia is probably the most common systemic finding of patients with solid tumors [56]. It is caused by either a functional iron deficiency (anemia of inflammation or chronic disease) or by iron sequestration. It is worth noting that most anemia cases were found in association with advanced BCC. Chronic bleeding from the large erosive tumor surfaces of advanced BCC could be a plausible anemia explanation; however, in two of the four KSC-associated cases (two of three BCC associated cases) the etiology of anemia was the rather rare pure red cell aplasia syndrome [57].

Bazex syndrome (acrokeratosis paraneoplastica), a rare obligate cutaneous PNS, [58] was the third more frequently reported PNS in association with a KSC. It is worth noting that although most cases in the literature are related to SCC of upper aerodigestive mucosae, cases compiled here are associated with cSCC of the extremities: a lower leg and a forearm tumor with axillary lymph node metastases.

A limitation of this review is the restriction of the literature source to Medline inclusions. However, this has most probably not affected the above conclusions since the compiled data set is rather representative of the topic.

## 5. Conclusions

Overall, patients with cSCC seem to be at a much higher risk to develop a PNS compared to BCC patients. Particularly, hypercalcemia in the setting of malignancy, related to increased PTHrP serum levels, was the most frequently reported PNS. It was seen almost exclusively in association with a cSCC. Physicians who care for patients with KSC should be alerted for this condition and adequately monitor susceptible patients to prevent a hypercalcemic crisis, an emergency condition which is associated with severe neurologic symptoms, poor prognosis, and decreased survival.

Taken together, descriptions of cases of KSC-associated PNS are rather scarcely reported in the medical literature. This is in distinct contrast with the high incidence of KSC, at least among Caucasians. Future studies should inquire whether KSC-associated PNS are truly rare events or whether they are simply underrecognized and consequently underreported conditions. In this context it could be interesting to compare the epidemiology of PNS in KSC with that of the other, non-keratinocyte derived skin cancers and also with keratinocyte tumors that originate from skin-adjacent mucosal surfaces (oropharynx, external genitalia). Nevertheless, we would like to predict that with the wider availability and application of the emerging treatment modalities for advanced KSC, the need to differentiate between adverse events and PNS will probably lead to increasing numbers of PNS observations in the future.

## Figures and Tables

**Table 1:** Characteristics of patients with a keratinocyte skin cancer associated with paraneoplastic syndromes.

Ref ^a^	Tumor	PNS ^b^	Age/Sex	Localization	History	Recurrence/Metastases ^c^	Timing of PNS ^d^	Tumor/PNS Treatment	Resolution of PNS
[9]	SCC	Bazex Syndrome	69/M	Axilla (metastasis)	SCC ipsilateral forearm	No/Yes	Before	Chemothe-rapy	Yes
[10]	SCC	Inflammatory arthralgias	52/M	Skin (area N/A)	N/A	No/No	After	Surgery	Yes
[11]	SCC	Neuropathy	50/M	Perineal	Hidradenitis suppurativa	No/No	Before	Surgery	Yes
[12]	SCC	Hypercalcemia	67/M	Chest wall	Semi-comatose patient	No/No	Simultane-ously	Patient refused therapy	No
[13]	SCC	Hypercalcemia, leukocytosis	58/M	Sole	Scar (accident)	No/Yes	After	Surgery, chemothe-rapy, radiation	Yes
[14]	SCC	Hypercalcemia	82/M	Finger	Excision 2 years ago	Yes/Yes	After	Surgery, chemothe-rapy, radiation	No (patient died)
[15]	SCC	Bazex Syndrome	54/M	Leg	Old scar	No/No	After	Surgery	Yes
[16]	SCC	Hypercalcemia	38/M	Axilla	SCC on arm (burn scar area)	Yes/Yes	After	Surgery	Yes
[17]	SCC	Hypercalcemia, leukocytosis	50/M	Sacral	Hidradenitis suppurativa	No/Yes	Simultane-ously	Chemo-therapy	No
[17]	SCC	Hypercalcemia	60/M	Hand	N/A	No/Yes	After	Chem-otherapy	No
[18]	SCC	Hypercalcemia, leukocytosis	20/M	Foot	Recessive dystrophic EB	Yes/Yes	Simultane-ously	Surgery, zolendro-nate	Yes
[19]	SCC	Hypercalcemia	74/M	Back	Porokerato-sis Mibelli	Yes/Yes	After	Surgery	Yes
[20]	SCC	Hypercalcemia	65/M	Buttock	Hidradenitis Suppurativa	Yes/Yes	After	Surgery, radiotherapy	Yes
[21]	SCC	Hypercalcemia	72/M	Leg	Chronic venous ulcer	No/No	Simultane-ously	Surgery	No
[22]	SCC	Hypercalcemia	45/M	Sacral	N/A	No/Yes	Simultane-ously	Cetuximab	No
[23]	SCC	Hypercalcemia	59/M	Scapular area	‘Pyoderma chronica’	Yes/No	After	Chemo-therapy, radiation	Yes
[24]	SCC	Hypercalcemia	65/F	Back	Lethargy	No/Yes	Simultane-ously	Chemo-therapy	Yes
[25]	SCC	Hypercalcemia	80/F	Leg	Thrombo-phlebitis	No/No	Simultane-ously	Surgery	No (patient died)
[26]	SCC	Hypercalcemia	17/M	Leg	Epidermo-lysis bullosa	Yes/Unknown	After	Denosumab	Yes
[27]	SCC	Hypercalcemia	38/M	Back	Burn scar	No/No	Simultane-ously	Surgery	Yes
[28]	SCC	Hypercalcemia	68/F	Arm	N/A	No/No	Simultane-ously	Surgery	Yes
[29]	BCC	Antiphospho-lipid syndrome	64/F	Face	Chronic heart failure	No/No	After	Surgery	Yes
[30]	BCC	Pure red cell aplasia	65/M	Shoulder	Hyper-tension	Yes/Yes	After	Surgery, radiatio,-chemo-therapy	No
[31]	TRC	Hypercalcemia	42/M	Scalp	Burn scar	No/No	Simultane-ously	Surgery	Yes
[32]	PLM	Hypercalcemia	32/M	Neck	N/A	No/No	Simultane-ously	Surgery	Yes
[33]	SCC	Pure Red Cell Aplasia	80/M	Ear	Benign Prostate Hypertrophy	No/Unknown	Before	Surgery	No
[34]	SCC	Hypercalcemia leucocytosis	5/F	Face	Xeroderma pigmento-sum	No/No	Simultane-ously	Chemo-therapy	Yes
[35]	SCC	Hypercalcemia	83/M	Retro-Ear	N/A	No/Yes	Simultane-ously	Radio-therapy	Yes
[36]	SCC	Hypercalcemia	35/M	Buttock	Hidradenitis suppurativa	No/No	Simultane-ously	Chemo-therapy	N/A
[37]	SCC	Hypercalcemia	81/M	Chest wall	BCC	No/No	Simultane-ously	Surgery	Yes
[38]	SCC	Hypercalcemia	50/M	Hip	Hidradenitis suppurativa	No/Yes	Simultane-ously	Surgery	Yes
[39]	SCC	Hypercalcemia, leukocytosis	45/M	Thigh	Burn scar	Yes/Yes	Simultane-ously	Chemo-therapy	Yes
[40]	SCC	Hypercalcemia	62/M	Groin	Psoriasis/chronic arsenic ingestion	No/No	After	Surgery	N/A
[41]	BCC	Anaemia	50/M	Chest wall	N/A	N/A/No	After	Surgery	Yes
[42]	BCC	Anaemia	76/F	Face	N/A	N/A	Simultane-ously	Radio-therapy	N/A
[43]	SCC	Hypercalcemia	53/M	Back	N/A	N/A/Yes	Simultane-ously	Chemo-therapy	No (patient died)
[44]	SCC	Hypercalcemia	68/M	Buttocks	Hidradenitis suppurativa	Yes/N/A	After	Chemo-therapy	No (patient died)
[45]	SCC	Hypercalcemia	63/M	Buttocks	Hidradenitis suppurativa	N/A	Simultane-ously	Surgery	No (patient died)
[46]	SCC	Hypercalcemia	51/M	Buttocks	Hidradenitis suppurativa	Yes/No	After	None specific	No (patient died)
[48]	SCC	Hypercalcemia	28/F	Ischial tuberosity	Paraplegia/-chronic decubitus ulcer	No/Yes	Simultane-ously	Zolendronic acid	Yes
[49]	SCC	Hypercalcemia	58/M	Leg	Lymph-edema	No/No	Simultane-ously	Surgery	Yes

The background color highlights the data that refer to one case. ^a^ Ref: References list number. ^b^ Abreviations. F: female; KSC: Keratinocyte Skin Cancer; M: male; N/A: information not available; PLM: pilomatricoma; PNS:paraneoplastic syndrome; SCC: cutaneous Squamous Cell Carcinoma; TRC: trichilemmal carcinoma. ^c^ Recurrence: local relapse; metastasis: distant relapse. ^d^ Timing of PNS. before: PNS diagnosis precedes tumor diagnosis; simultaneous: PNS present at the time of tumor diagnosis; after: PNS diagnosis after tumor diagnosis.

**Table 2:** Paraneoplastic syndromes in patients with keratinocyte skin cancer. Compilation of the clinical features of all cases and separately for the different skin cancer types. No (%): Number of cases and % of corresponding cases with available information.

Keratinocyte Skin Cancer (No)		Total (N = 41)	BCC ^a^ (N = 4)	SCC (N = 35)	Other ^b^ (N = 2)
**Sex, No (%)**	Male	34 (83)	2 (50)	30 (86)	2 (100)
	Female	7 (17)	2 (50)	5 (14)	0 (0)
**Localization ^c^, No (%)**	Head/neck	7 (18)	2 (50)	3 (9)	2 (100)
	Trunk	19 (47)	2 (50)	17 (50)	0
	Extremities	14 (35)	0	14 (41)	0
	N/A	1	0	1	0
**PNS, No (%)**	MAH/HHM	32 (78)	0 (0)	30 (85)	2 (100)
	Anemia	4 (10)	3 (75)	1 (3)	0 (0)
	Bazex syndrome	2 (5)	0 (0)	2 (6)	0 (0)
	Other ^i^	3 (7)	1 (25)	2 (6)	0 (0)
**Timing of PNS ^d^, No (%)**	before	3 (7)	0 (0)	3 (9)	0 (0)
	simultaneously	22 (54)	1 (25)	19 (54)	2 (100)
	after	16 (39)	3 (75)	13 (37)	0 (0)
**Primary ^e^, No (%)**	No	21 (55)	1 (33)	20 (61)	0 (0)
	Yes	17 (45)	2 (67)	13 (39)	2 (100)
	N/A	3	1	2	0
**Recurrence ^f^, No (%)**	No	27 (71)	2 (67)	23 (70)	2 (100)
	Yes	11 (29)	1 (33)	10 (30)	0 (0)
	N/A	3	1	2	0
**Metastases, No (%)**	No	20 (54)	3 (75)	15 (48)	2 (100)
	Yes	17 (46)	1 (25)	16 (52)	0 (0)
	N/A	4	0	4	0
**Predilection ^g^, No (%)**	No	9 (28)	2 (100)	7 (24)	0 (0)
	Yes	23 (72)	0	22 (76)	1 (100)
	N/A	9	2	6	1
	Genoderma-tosis ^j^	4 (17)	0 (0)	4 (18)	0 (0)
	Acquired ^k^	19 (83)	0 (0)	18 (82)	1 (100)
	*Scar/Ulcus*	*9 (48)*	*0 (0)*	*8 (44)*	*1 (100)*
	*Hidradenitis suppurativa*	*8 (42)*	*0 (0)*	*8 (44)*	*0 (0)*
	*Other ^l,m^*	*2 (10)*	*0 (0)*	*2 (12)*	*0 (0)*
**Resolution ^h^, No (%)**	Yes	25 (66)	2 (67)	21 (64)	2 (100)
	No	13 (34)	1 (33)	12 (36)	0 (0)
	N/A	3	1	2	0

The background color highlights the data that refer to one factor. ^a^ Abbreviations. BCC: basal cell carcinoma, SCC: cutaneous squamous cell carcinoma, N/A: not available, PNS: paraneoplastic syndrome, MAH: malignancy associated hypercalcemia, HHM: humoral hypercalcemia of malignancy. ^b^ One case of trichilemal carcinoma and one case of pilomatrixoma. ^c^ Localization: localization of the primary neoplasm. ^d^ Timing of PNS: time point of PNS diagnosis relative to the diagnosis of skin cancer. ^e^ Primary: primary tumor only. ^f^ Recurrence: local tumor recurrence after treatment. ^g^ Predilection: presence of a condition that predisposes to skin cancer development. ^h^ Resolution: resolution of paraneoplastic syndrome after skin cancer treatment. ^I^ Inflammatory arthralgias, neuropathy, antiphospholipid syndrome. ^j^ Genodermatoses: recessive dystrophic epidermolysis bullosa, porokeratosis Mibelli, xeroderma pigmentosum. ^k^ Acquired: acquired predilection. ^l^ Chronic arsenic intoxication, lymphedema. ^m^Italics denote the subanalysis of cases with an acquired predilection.

**Table 3:** Malignancy associated hypercalcemia in KSC patients: Core demographic and laboratory findings. No (%): Number (% cases with available data).

Demographics		
**Age, median [range], y**		58 [5–83]
**Sex, No (%)**	Women	5 (16)
	Men	27 (84)
**Tumor, No (%)**	SCC	30 (94)
	Other	2 (6)
**Laboratory findings**		
**Hypercalcemia ^a^** **, Νο (%)**	mild	4 (13)
	moderate	9 (28)
	severe	19 (59)
**Phosphorus ^b^** **, Νο (%)**	decreased	5 (25)
	norm	14 (70)
	increased	1 (5)
	N/A	12 (38)
**Parathormone ^b^** **, PTH, Νο (%)**	decreased	15 (54)
	norm	13 (46)
	increased	0 (0)
	N/A	4 (12)
**Parathormone related protein, PTHrP ^b^,** **Νο** **(%)**	decreased	0 (0)
	norm	1 (5)
	increased	20 (95)
	N/A	11 (34)
**Vitamin D_3_** **^b^, No (%)**	decreased	5 (31)
	norm	10 (63)
	increased	1 (6)
	N/A	16 (50)

The background color highlights the data that refer to one factor. ^a^ Mild: 10.7—11.9 mg/dL; moderate: 12.0–13.9 mg/dL; severe: ≥14 mg/dL. ^b^ Explanations. norm: within normal range; increased: above norm range; decreased: below norm range; N/A: value not available. Abbreviations. D_3_: 1,25 (OH)_2_ D_3_; KSC: Keratinocyte Skin Cancer; PLM: pilomatrixoma; PTH: parathormone; PTHrP: parathormone related protein; SCC: cutaneous Squamous Cell Carcinoma; TRC: trichilemmal carcinoma.

**Table 4:** Age of patients (Median [Range]) at the time of diagnosis of a keratinocyte skin cancer associated paraneoplastic syndrome, stratified according to patients’ gender.

Factor		Total (N = 41)	Male (N = 34)	Female (N = 7)
**Tumor ^a^**	BCC (N = 4)	64.5 [50–76]	57.5 [50–64]	70.0 [64–76]
	SCC (N = 35)	58.0 [5–83]	58.0 [17–83]	66.5 [05–80]
	Other ^b^ (N = 2)	37.0 [32–42] *	37.0 [32–42] *	-
**PNS**	HHM all (N = 32)	51.0 [5–83]	58.0 [17–83]	65.0 [5–80]
	*HHM without Leukocytosis (N = 27)*	*60.0 [17–83]*	*59.0 [17–83]*	*66.5 [5–80]*
	*HHM with Leukocytosis (N = 5)*	*45.0 [5–58]*	*47.5 [20–58]*	5 *
	Other than HHM (N = 9)	64.0 [50–80]	54.0 [50–80]	64 *
	*Anemia (N = 4)*	*70.5 [50–80]*	*65.0 [50–80]*	76 *
	*Bazex syndrome (N = 2)*	61.5 [54–69] *	61.5 [54–69] *	*-*
	*Other ^c^ (N = 3)*	*52.0 [50–64]*	*51.0 [50–52]*	64 *
**Localization ^d^**	Head/Neck (N = 7)	64.0 [5–83]	61.0 [32–83]	64.0 [5–76]
	Trunk (N = 20)	56.0 [28–81]	59.0 [28–81]	28 *
	Extremities (N = 13)	58.0 [17–82]	56.0 [17–82]	68.0 [65–68]
	N/A (N = 1)	52 *	52 *	-
**Predilection ^e^**	No (N = 9)	74.5 [64–82]	67.0 [65–82]	65.0 [64–80]
	Yes (N = 23)	50.0 [5–74]	52.5 [17–74]	5 *
	N/A (N = 9)	62.5 [32–83]	52.0 [32–83]	72.0 [68–76]
	Genodermatosis (N = 4)	18.5 [5–74]	20.0 [17–74]	5 *
	Acquired ^f^ (N = 19)	51.0 [28–72]	58.0 [35–72]	28 *
	*Scar/ulcus (N = 9)*	*45.0 [28–72]*	*52.5 [38–72]*	28 *
	*Hidradenitis suppurativa (N = 8)*	*50.5 [35–68]*	*50.0 [35–68]*	-
	*Other ^g^ (N = 2)*	60.0 [58–62] *	69.0 [62–74] *	-
**Resolution ^h^**	No (N = 13)	65.0 [45–82]	63.0 [45–82]	72.5 [65–80]
	Yes (N = 25)	52.0 [5–83]	52.0 [17–83]	46.0 [5–68]
	N/A (N = 3)	62.5 [35–76]	48.5 [35–62] *	76 *

The different colors separate rows referring to the different factor. Italics indicate data referring to subcategories of factors. ^a^ Abbreviations. BCC: basal cell carcinoma, SCC: cutaneous squamous cell carcinoma, HHM: humoral hypercalcemia of malignancy, PNS: paraneoplastic syndrome. ^b^ Trichilemmal carcinoma, pilomatrixoma. ^c^ Inflammatory arthralgias, neuropathy, antiphospholipid syndrome. ^d^ Localization: localization of the primary neoplasm. ^e^ Predilection: presence of a condition that predisposes to skin cancer development. ^f^ Acquired: acquired predilection. ^g^ Chronic arsenic intoxication, lymphedema. ^h^ Resolution: resolution of paraneoplastic syndrome after skin cancer treatment. * For <3 cases single values instead of ‘range’ are displayed.

## Data Availability

Not applicable.

## Supplementary Materials

The following are available online at https://www.mdpi.com/article/10.3390/cancers14010249/s1, Table S1: Paraneoplastic syndromes in patients with keratinocyte skin cancer: Compilation of the clinical features stratified according to patients’ gender, Table S2: Paraneoplastic syndromes in patients with keratinocyte skin cancer. Compilation of the clinical features stratified according to the different skin cancer types. No (%): Number of cases and distribution of the cases among the different cancer types (% of cases with available information), Table S3: Malignancy associated hypercalcemia (MAH) in KSC patients: Per patient laboratory findings of the N = 32 cases with MAH.

## References

[1] Hobbs C.B., Miller A.L. (1966). Review of Endocrine Syndromes Associated with Tumours of Non-Endocrine Origin. J. Clin. Pathol..

[2] Bilynsky B.T., Dzhus M.B., Litvinyak R.I. (2015). The Conceptual and Clinical Problems of Paraneoplastic Syndrome in Oncology and Internal Medicine. Exp. Oncol..

[3] Dimitriadis G.K., Angelousi A., Weickert M.O., Randeva H.S., Kaltsas G., Grossman A. (2017). Paraneoplastic Endocrine Syndromes. Endocr.-Relat. Cancer.

[4] Maverakis E., Goodarzi H., Wehrli L.N., Ono Y., Garcia M.S. (2012). The Etiology of Paraneoplastic Autoimmunity. Clin. Rev. Allergy Immunol..

[5] Thiers B.H., Sahn R.E., Callen J.P. (2009). Cutaneous Manifestations of Internal Malignancy. CA Cancer J. Clin..

[6] Ehst B.D., Minzer-Conzetti K., Swerdlin A., Devere T.S. (2010). Cutaneous Manifestations of Internal Malignancy. Curr. Probl. Surg..

[7] Vyas R., Selph J., Gerstenblith M.R. (2016). Cutaneous Manifestations Associated with Melanoma. Semin. Oncol..

[8] Iyer J.G., Parvathaneni K., Bhatia S., Tarabadkar E.S., Blom A., Doumani R., McKenzie J., Asgari M.M., Nghiem P. (2016). Paraneoplastic Syndromes (PNS) Associated with Merkel Cell Carcinoma (MCC): A Case Series of 8 Patients Highlighting Different Clinical Manifestations. J. Am. Acad. Derm..

[9] Vatandoust S., McKay B.P., McLeay W., Miliauskas J., Gordon L., Wesley J.A., Kichenadasse G. (2016). Acrokeratosis Paraneoplastica (Bazex Syndrome) Associated with Metastatic Cutaneous Squamous Cell Carcinoma. Intern. Med. J..

[10] Hakkou J., Rostom S., Bahiri R., Hajjaj-Hassouni N. (2012). Paraneoplastic rheumatic syndromes: Report of eight cases and review of literature. Rheumatol. Int..

[11] Rosenzweig L.B., Brett A.S., Lefaivre J.-F., Vandersteenhoven J.J. (2005). Hidradenitis Suppurativa Complicated by Squamous Cell Carcinoma and Paraneoplastic Neuropathy. Am. J. Med. Sci..

[12] Cisneros G., Lara L.F., Crock R., Whittier F.C. (2001). Humoral hypercalcemia of malignancy in squamous cell carcinoma of the skin: Parathyroid hormone-related protein as a cause. South Med. J..

[13] Kato N., Yasukawa K., Onozuka T., Kimura K. (1999). Paraneoplastic syndromes of leukocytosis, thrombocytosis, and hypercalcemia associated with squamous cell carcinoma. J. Dermatol..

[14] Mori H., Aoki K., Katayama I., Nishioka K., Umeda T. (1996). Humoral Hypercalcemia of Malignancy with Elevated Plasma PTHrP, TNF Alpha and IL-6 in Cutaneous Squamous Cell Carcinoma. J. Derm..

[15] Hara M., Hunayama M., Aiba S., Suetake T., Watanabe M., Tanaka M., Tagami H. (1995). Acrokeratosis paraneoplastica (Bazex syndrome) associated with primary cutaneous squamous cell carcinoma of the lower leg, vitiligo and alopecia areata. Br. J. Dermatol..

[16] Gerner R.E., Moore G.E. (1974). Burn Scar Carcinoma and Hypercalcemia. Ann. Surg..

[17] Ben Said B., Maitre S., Perrot J.-L., Labeille B., Cambazard F. (2010). Syndrome paranéoplasique hypercalcémie–hyperleucocytose au cours des carcinomes épidermoïdes cutanés. À propos de deux observations. La Rev. De Méd. Interne.

[18] Miura K., Umegaki N., Kitaoka T., Kubota T., Namba N., Etani Y., Hirai H., Kogaki S., Nakajima S., Takahashi Y. (2011). A male patient with humoral hypercalcemia of malignancy (hhm) with leukocytosis caused by cutaneous squamous cell carcinoma resulting from recessive dystrophic epidermolysis bullosa. Clin. Pediatr. Endocrinol..

[19] Sawai T., Hayakawa H., Danno K., Miyauchi H., Uehara M. (1996). Squamous cell carcinoma arising from giant porokeratosis: A case with extensive metastasis and hypercalcemia. J. Am. Acad. Dermatol..

[20] Miquel J., Adamski H., Faujour G. (2009). Hypercalcémie aiguë et carcinomes épidermoïdes multiples compliquant une hidradénite suppurée. Presse Med..

[21] Crespo M., Sopeña B., Orloff J.J., CameselleTeijeiro J.F., Dann P., Andrade M.A., Freire M., de la Fuente J., Martinez-Vazquez C. (1999). Immunohistochemical detection of parathyroid hormone-related protein in a cutaneous squamous cell carcinoma causing humoral hypercalcemia of malignancy. Arch Pathol Lab Med..

[22] O’Malley J.T., Schoppe C., Husain S., Grossman M.E. (2014). Squamous cell carcinoma (Marjolin’s ulcer) arising in a sacral decubitus ulcer resulting in humoral hypercalcemia of malignancy. Case Rep. Med..

[23] Ito Y., Iwasaki T., Wakamatsu K., Hashimoto M., Iizuka H. (2009). Hypercalcemia associated with squamous cell carcinoma arising in pyoderma chronica. J. Dermatol..

[24] Kaur M.R., Marsden J.R., Nelson H.M. (2007). Humoral hypercalcaemia of malignancy associated with primary cutaneous squamous cell carcinoma. Clin. Exp. Dermatol..

[25] Loche F., Bennet A., Bazex J., Thouvenin M.D. (1999). Hypercalcaemia of malignancy associated with invasive cutaneous squamous cell carcinoma. Br. J. Dermatol..

[26] Giri D., Ramakrishnan R., Hayden J., Brook L., Das U., Mughal M.Z., Selby P., Dharmaraj P., Senniappan S. (2015). Denosumab therapy for refractory hypercalcemia secondary to squamous cell carcinoma of skin in epidermolysis bullosa. World J. Oncol..

[27] Sungur N., Kilinç H., Uysal C., Ortak T., Sensöz O. (2001). A gigantic squamous cell carcinoma with hypercalcemia arising in an old burn scar. Ann. Plast. Surg..

[28] Marino M.T., Asp A.A., Budayer A.A., Marsden J.S., Strewler G.J. (1993). Hypercalcaemia and Elevated Levels of Parathyroid Hormone-Related Protein in Cutaneous Squamous/Basal Cell Carcinoma. J. Intern. Med..

[29] Funauchi M., Yamagata T., Sugiyama M., Ikoma S., Sakaguchi M., Kinoshita K., Kawata A. (2007). A Case of Antiphospholipid Antibody Syndrome That Manifested in the Course of Basal Cell Carcinoma. Mod. Rheumatol..

[30] Carneiro B.A., Watkin W.G., Mehta U.K., Brockstein B.E. (2006). Metastatic basal cell carcinoma: Complete response to chemotherapy and associated pure red cell aplasia. Cancer Invest..

[31] Ikeda T., Tsuru K., Hayashi K., Ichihashi M., Ueda M. (2000). Hypercalcemia of malignancy associated with trichilemmal carcinoma in burn scar. Acta Derm Venereol..

[32] Yamauchi M., Yotsuyanagi T., Saito T., Ikeda K., Urushidate S., Higuma Y. (2010). Three Cases of Giant Pilomatrixoma--Considerations for Diagnosis and Treatment of Giant Skin Tumours with Abundant Inner Calcification Present on the Upper Body. J. Plast. Reconstr. Aesthet. Surg..

[33] Guthrie T.H., Thornton R.M. (1983). Pure red cell aplasia obscured by a diagnosis of carcinoma. South Med. J..

[34] Emir S., Hacısalihoğlu Ş., Özyörük D., Kaçar D., Erdem A., Karakuş E. (2017). Squamous cell carcinoma associated with xeroderma pigmentosum: An unusual presentation with a tremendously huge mass over the face and paraneoplastic hypercalcemia-hyperleukocytosis. Turk J. Pediatr..

[35] Vlachostergios P.J., Balmiki R.L. (2014). Bone metastases and hypercalcaemia from cutaneous squamous cell carcinoma. BMJ Case Rep..

[36] Sparks M.K., Kuhlman D.S., Prieto A., Callen J.P. (1985). Hypercalcemia in association with cutaneous squamous cell carcinoma. Occurrence as a late complication of hidradenitis suppurativa. Arch Dermatol..

[37] Picascia D.D., Caro W.A. (1987). Cutaneous Squamous Cell Carcinoma and Hypercalcemia. J. Am. Acad. Derm..

[38] Welsh D.A., Powers J.S. (1993). Elevated parathyroid hormone-related protein and hypercalcemia in a patient with cutaneous squamous cell carcinoma complicating hidradenitis suppurativa. South Med. J..

[39] Hayakawa Y., Ishizaki H., Tanabe S., Kimura A., Yamamichi N. (1986). Squamous cell carcinoma with hypercalcemia and leukocytosis. Dermatologica.

[40] Southwick G.J., Schwartz R.A. (1979). Arsenically Associated Cutaneous Squamous Cell Carcinoma with Hypercalcemia. J. Surg. Oncol..

[41] Clements W.D., Ritchie A.J., Kinley J.G. (1991). Basal cell carcinoma presenting with profound anaemia. Ulster Med. J..

[42] Higgins J., Hull S.M. (1996). Profound anaemia secondary to an ulcerated basal cell carcinoma. Clin. Exp. Dermatol..

[43] Reynaud-Mendel B., Robert C., Flageul B., de Vernejoul M.C., Verola O., Dubertret L. (1997). Malignant hypercalcemia induced by a parathyroid hormone-related protein secreted by a cutaneous squamous cell carcinoma. Arch Dermatol..

[44] Constantinou C., Widom K., Desantis J., Obmann M. (2008). Hidradenitis suppurativa complicated by squamous cell carcinoma. Am. Surg..

[45] Pitch M.A., Bryan D.J., McMillan J., Chavez L., Hammes S.R., Scott G., Mercurio M.G., Somers K.E. (2018). A Fatal Case of Parathyroid Hormone-Related Peptide (PTHrP)-Producing Squamous Cell Carcinoma Arising in the Context of Long-Standing Hidradenitis Suppurativa. JAAD Case Rep..

[46] Makoui C., Fishburne C. (1978). Hypercalcemia in squamous cell carcinoma of the skin. JAMA.

[47] Nicolae I., Schipor S. (2010). PTH-Independent Hypercalcaemia and Non-Melanoma Skin Cancer. J. Eur. Acad. Derm. Venereol..

[48] Ramadas P., Bansal N., Krishnan P., Caza T., Manocha D. (2016). Hypercalcemia as the first diagnostic clue of a malignant decubitus ulcer. Austin Intern. Med..

[49] Saeed M.A., George G.A., Lajara S., Perkins B., Knight C.M. (2017). A Rare Case of Hypercalcemic Crisis Secondary to a Squamous Cell Carcinoma Arising from a Nonhealing Ulcer. AACE Clin. Case Rep..

[50] Goltzman D., Feingold K.R., Anawalt B., Boyce A., Chrousos G., de Herder W.W., Dhatariya K., Dungan K., Grossman A., Hershman J.M. (2000). Approach to Hypercalcemia. Endotext.

[51] Zagzag J., Hu M.I., Fisher S.B., Perrier N.D. (2018). Hypercalcemia and Cancer: Differential Diagnosis and Treatment. CA Cancer J. Clin..

[52] Stewart A.F. (2005). Clinical Practice. Hypercalcemia Associated with Cancer. N. Engl. J. Med..

[53] Weckmann M.T., Gröne A., Capen C.C., Rosol T.J. (1997). Regulation of Parathyroid Hormone-Related Protein Secretion and MRNA Expression in Normal Human Keratinocytes and a Squamous Carcinoma Cell Line. Exp. Cell Res..

[54] Burt M.E., Brennan M.F. (1980). Incidence of Hypercalcemia and Malignant Neoplasm. Arch. Surg..

[55] Attanoos R.L., Appleton M.A., Hughes L.E., Ansell I.D., Douglas-Jones A.G., Williams G.T. (1993). Granulomatous Hidradenitis Suppurativa and Cutaneous Crohn’s Disease. Histopathology.

[56] Gaspar B.L., Sharma P., Das R. (2015). Anemia in Malignancies: Pathogenetic and Diagnostic Considerations. Hematology.

[57] Means R.T. (2016). Pure Red Cell Aplasia. Blood.

[58] Räßler F., Goetze S., Elsner P. (2017). Acrokeratosis Paraneoplastica (Bazex Syndrome)—A Systematic Review on Risk Factors, Diagnosis, Prognosis and Management. J. Eur. Acad. Derm. Venereol..

