# Supplementary material for: Paraneoplastic Syndromes in Patients with Keratinocyte Skin Cancer
Source: Cancers (Basel). 2022 Jan 4;14(1):249. doi: 10.3390/cancers14010249 (PMC8750284; doi:10.3390/cancers14010249)
Supplement: Supplementary file 1 [file cancers-14-00249-s001.zip › cancers-1438590-supplementary.pdf]

Supplementary Material
Paraneoplastic Syndromes in Patients with Keratinocyte Skin Cancer

Christoforos Vlachos, Chrysanthi Tziortzioti and Ioannis D. Bassukas

Content

Table S1 p. 2

Table S2 p. 3

Table S3 p. 5

Table S1. Paraneoplastic syndromes in patients with keratinocyte skin cancer: Compilation of the clinical features stratified according to patients' gender.

Keratinocyte skin cancer		Total (N=41)	Male (N=34)	Female (N=7)
**Localization[a]**	Head / neck	7	4	3
	Trunk	19	17	2
	Extremities	14	12	2
	N/A	1	1	0
**PNS**	MAH / HHM	32	27	5
	Anemia	4	3	1
	Bazex syndrome	2	2	0
	Other[i]	3	2	1
**Timing of PNS[b]**	before	3	3	0
	simultaneously	22	16	6
	after	16	15	1
**Primary[c]**	No	21	15	3
	Yes	17	15	2
	N/A	3	4	1
**Recurrence[d]**	No	27	21	6
	Yes	11	11	0
	N/A	3	2	1
**Metastases**	No	20	16	4
	Yes	17	15	2
	N/A	4	3	1
**Predilection[e]**	No	9	5	4
	Yes	23	21	2
	N/A	9	7	2
	Genodermatosis[g]	4	3	1
	Acquired[h]	19	18	1
	Scar/Ulcus	9	8	1
	(burn) Scar	*6*	*6*	*0*
	Ulcus	*3*	*2*	*1*
	Hidradenitis suppurativa	8	8	0
	Chronic arsenic intoxication	1	1	0
	Lymphedema	1	1	0
**Resolution[f]**	Yes	25	20	5
	No	13	12	1
	N/A	3	2	1

Abbreviations. N/A: not available, PNS: paraneoplastic syndrome, MAH: malignancy associated hypercalcemia, HHM: humoral hypercalcemia of malignancy.

[a]Localization: localization of the primary neoplasm.

[b]Timing of PNS: time point of PNS diagnosis relative to the diagnosis of skin cancer.

[c]Primary: primary tumor only

[d]Recurrence: local tumor recurrence after treatment.

[e]Predilection: Presence of a condition that predisposes to skin cancer development.

[f]Resolution: resolution of paraneoplastic syndrome after skin cancer treatment. inflammatory arthralgias, neuropathy, antiphospholipid syndrome

[g]Genodermatoses: recessive dystrophic epidermolysis bullosa, porokeratosis Mibelli, xeroderma pigmentosum

[h]Acquired: acquired predilection.

Table S2. Paraneoplastic syndromes in patients with keratinocyte skin cancer. Compilation of the clinical features stratified according to the different skin cancer types. No (%): Number of cases and distribution of the cases among the different cancer types (% of cases with available information).

Keratinocyte skin cancer, No (%)		Total 41(100)	BCC[a] 4(10)	SCC 35(85)	Other[b] 2(5)
**Sex, No (%)**	Male	34	2 (6)	30 (88)	2 (6)
	Female	7	2 (29)	5 (71)	0 (0)
**Localization[c], No (%)**	Head / neck	7	2 (29)	3 (42)	2 (29)
	Trunk	19	2 (11)	17 (89)	0 (0)
	Extremities	14	0 (0)	14 (100)	0 (0)
	N/A	1	0	1	0
**PNS, No (%)**	MAH / HHM	32	0 (0)	30 (94)	2 (6)
	Anemia	4	3 (75)	1 (25)	0 (0)
	Bazex syndrome	2	0 (0)	2 (100)	0 (0)
	Other[i]	3	1 (33)	2 (67)	0 (0)
**Timing of PNS[d], No (%)**	before	3	0 (0)	3 (100)	0 (0)
	simultaneously	22	1 (5)	19 (85)	2 (10)
	after	16	3 (19)	13 (81)	0 (0)
**Primary[e], No (%)**	No	21	1 (5)	20 (95)	0 (0)
	Yes	17	2 (12)	13 (86)	2 (12)
	N/A	3	1	2	0
**Recurrence[f], No (%)**	No	27	2 (7)	23 (86)	2 (7)
	Yes	11	1 (9)	10 (91)	0 (0)
	N/A	3	1	2	0
**Metastases, No (%)**	No	20	3 (15)	15 (75)	2 (10)
	Yes	17	1 (6)	16 (94)	0 (0)
	N/A	4	0	4	0
**Predilection[g], No (%)**	No	9	2 (22)	7 (78)	0 (0)
	Yes	23	0 (0)	22 (96)	1 (4)
	N/A	9	2	6	1
	Genodermatosis[j]	4	0 (0)	4 (100)	0 (0)
	Acquired[k]	19	0 (0)	18 (95)	1 (5)
	Scar/Ulcus	*9*	*0 (0)*	*8 (89)*	*1 (11)*
	Hidradenitis suppurativa	*8*	*0 (0)*	*8 (100)*	*0 (0)*
	Other[l]	*2*	*0 (0)*	*2 (100)*	*0 (0)*
**Resolution[h], No (%)**	Yes	25	2 (8)	21 (84)	2 (8)
	No	13	1 (8)	12 (92)	0 (0)
	N/A	3	1	2	0

[a]Abbreviations. BCC: basal cell carcinoma, SCC: cutaneous squamous cell carcinoma, N/A: not available, PNS: paraneoplastic syndrome, MAH: malignancy associated hypercalcemia, HHM: humoral hypercalcemia of malignancy.

[b]One case of trichilemal carcinoma and one case of pilomatrixoma.

[c]Localization: localization of the primary neoplasm.

[d]Timing of PNS: time point of PNS diagnosis relative to the diagnosis of skin cancer.

[e]Primary: primary tumor only

[f]Recurrence: local tumor recurrence after treatment.

[g]Predilection: Presence of a condition that predisposes to skin cancer development.
[h]Resolution: resolution of paraneoplastic syndrome after skin cancer treatment.
[i]Inflammatory arthralgias, neuropathy, antiphospholipid syndrome
[j]Genodermatoses: recessive dystrophic epidermolysis bullosa, porokeratosis Mibelli, xeroderma pigmentosum
[k]Acquired: acquired predilection.
[l]Chronic arsenic intoxication, lymphedema.

Table S3. Malignancy associated hypercalcemia (MAH) in KSC patients: Per patient laboratory findings of the N=32 cases with MAH.

Ref[a]	Tumor	Age/Sex[b]	Hypercalcemia[c]	Phosphorus	PTH[d,e]	PTHrP[e]	D_3[e]
**12**	SCC	67/M	severe	increased	decreased	increased	decreased
**13**[f]	SCC	58/M	severe	N/A	decreased	N/A	N/A
**14**	SCC	82/M	moderate	norm	norm	increased	N/A
**16**	SCC	38/M	moderate	norm	norm	N/A	norm
**17**[f]	SCC	50/M	mild	N/A	norm	increased	N/A
**17**[g]	SCC	60/M	mild	N/A	N/A	N/A	N/A
**18**[f]	SCC	20/M	severe	norm	norm	increased	decreased
**19**	SCC	74/M	severe	norm	norm	N/A	decreased
**20**[g]	SCC	65/M	moderate	N/A	norm	norm	norm
**21**	SCC	72/M	moderate	N/A	norm	increased	norm
**22**	SCC	45/M	severe	N/A	decreased	increased	decreased
**23**	SCC	59/M	moderate	decreased	decreased	increased	N/A
**24**	SCC	65/F	severe	N/A	decreased	increased	N/A
**25**	SCC	80/F	severe	decreased	decreased	increased	norm
**26**	SCC	17/M	severe	norm	decreased	increased	increased
**27**	SCC	38/M	moderate	decreased	norm	N/A	norm
**28**	SCC	68/F	severe	norm	decreased	increased	N/A
**31**	TRC[d]	42/M	severe	N/A	N/A	increased	N/A
**32**	PLM[d]	32/M	severe	N/A	decreased	increased	norm
**34**[f]	SCC	5/F	severe	norm	decreased	N/A	N/A
**35**	SCC	83/M	moderate	decreased	decreased	increased	N/A
**36**[g]	SCC	35/M	severe	norm	norm	N/A	N/A
**37**	SCC	81/M	severe	norm	norm	N/A	N/A
**38**[g]	SCC	50/M	severe	norm	decreased	increased	norm
**39**[f]	SCC	45/M	severe	N/A	norm	N/A	N/A
**40**	SCC	62/M	severe	norm	N/A	N/A	N/A
**43**	SCC	53/M	moderate	decreased	decreased	increased	N/A
**44**[g]	SCC	68/M	moderate	N/A	N/A	N/A	N/A
**45**[g]	SCC	63/M	mild	norm	decreased	increased	norm
**46**[g]	SCC	51/M	mild	norm	norm	increased	norm
**48**	SCC	28/F	severe	N/A	norm	increased	norm
**49**	SCC	58/M	severe	norm	decreased	increased	decreased

[a]Ref: Reference number (References list in main text). [b]M: male, F: female. [c]Mild: 10.7 – 11.9 mg/dL; moderate: 12.0-13.9 mg/dL; severe: ≥14 mg/dL. [d]Abbreviations. D_3: 1,25 $(OH)_2$ D_3; KSC: Keratinocyte Skin Cancer; PLM: pilomatrixoma; PTH: parathormone; PTHrP: parathormone related protein; SCC: cutaneous Squamous Cell Carcinoma; TRC: trichilemmal carcinoma. [e]Explanations. norm: within normal range; increased: above norm range; decreased: below norm range; N/A: value not available. [f]Hypercalcemia-Leukocytosis cases. [g]Associated with hidradenitis suppurativa.
